# Smoking during Pregnancy Affects Speech-Processing Ability in Newborn Infants

**DOI:** 10.1289/ehp.9521

**Published:** 2006-11-28

**Authors:** Alexandra P.F. Key, Melissa Ferguson, Dennis L. Molfese, Kelley Peach, Casey Lehman, Victoria J. Molfese

**Affiliations:** 1 Kennedy Center for Research on Human Development and Department of Hearing and Speech Sciences, Vanderbilt University, Nashville, Tennessee, USA; 2 Department of Psychological and Brain Sciences, University of Louisville, Louisville, Kentucky, USA; 3 Birth Defects Center, Department of Molecular, Cellular and Craniofacial Biology, University of Louisville, Louisville, Kentucky, USA; 4 College of Education and Human Development, University of Louisville, Louisville, Kentucky, USA

**Keywords:** ERP, evoked potentials, newborn, prenatal, smoking, speech

## Abstract

**Background:**

Tobacco smoking during pregnancy is known to adversely affect development of the central nervous system in babies of smoking mothers by restricting utero–placental blood flow and the amount of oxygen available to the fetus. Behavioral data associate maternal smoking with lower verbal scores and poorer performance on specific language/auditory tests.

**Objectives:**

In the current study we examined the effects of maternal smoking during pregnancy on newborns’ speech processing ability as measured by event-related potentials (ERPs).

**Method:**

High-density ERPs were recorded within 48 hr of birth in healthy newborn infants of smoking (*n* = 8) and nonsmoking (*n* = 8) mothers. Participating infants were matched on sex, gestational age, birth weight, Apgar scores, mother’s education, and family income. Smoking during pregnancy was determined by parental self-report and medical records. ERPs were recorded in response to six consonant–vowel syllables presented in random order with equal probability.

**Results:**

Brainwaves of babies of nonsmoking mothers were characterized by typical hemisphere asymmetries, with larger amplitudes over the left hemisphere, especially over temporal regions. Further, infants of nonsmokers discriminated among a greater number of syllables whereas the newborns of smokers began the discrimination process at least 150 msec later and differentiated among fewer stimuli.

**Conclusions:**

Our findings indicate that prenatal exposure to tobacco smoke in otherwise healthy babies is linked with significant changes in brain physiology associated with basic perceptual skills that could place the infant at risk for later developmental problems.

Cigarettes are among the nonmedicinal drugs used most widely during pregnancy (Fried et al. 1997), especially in Western cultures (Fried et al. 1997; Hardy and Mellits 1972; Makin et al. 1991; Weitzman et al. 2002). Although the general population of smokers is declining, pregnant women show the slowest rate of decline (Fried 2002; McCartney et al. 1994). Recent reports indicate that in the United States alone, 18.5% of all women smoke [Centers for Disease Control and Prevention (CDC) 2005], whereas 11.4% of women smoked during pregnancy (the rates for individual states were as high as 26%; CDC 2004).

Numerous studies report that maternal cigarette smoking during pregnancy may have a harmful effect on fetal development (Hellstrom-Lindahl and Nordberg 2002; Lassen and Oei 1998; McCartney et al. 1994; Oliff and Gallardo 1999). Both carbon monoxide and nicotine reduce the amount of oxygen available to fetal tissue by restricting the utero–placental blood flow (Naeye and Peters 1984). This can have potential negative effects on the central nervous system (Fried 2002) through cell damage and reduced cell number caused by errors in cell development (Roy and Sabherwal 1994) and a premature change from replication to differentiation (Slotkin 1998). These changes often occur at thresholds below those necessary for growth impairment and may not always manifest as intrauterine growth retardation [Slotkin 1998; see also Ernst et al. (2001) for review]. Nevertheless, low birth weight in newborns is the most consistently reported consequence of maternal smoking (Dejin-Karlsson et al. 1998; Fried 2002; Hardy and Mellits 1972; Lassen and Oei 1998).

Prenatal exposure to tobacco smoke is also linked to various health, behavioral, and cognitive impairments (see Weitzman et al. 2002 for review). Neonatal hyperactivity (greater excitability, heightened tremors and startles) is frequently noted among newborns prenatally exposed to tobacco (Fried et al. 1987; Law et al. 2003; Longo 1977). The American Academy of Pediatrics Committee on Environmental Health (1997) noted increased incidents of asthma, respiratory infections, and middle ear effusions. Recently, Wakschlag and Hans (2002) reported lower sociability/ negative emotionality during infancy and increased likelihood of conduct disorders during childhood in boys but not girls born to smoking mothers. Others observed higher frequencies of behavioral problems such as disruptive behavior, conduct disorder, and delinquency, including substance abuse, in both male and female children of smokers (Brennan et al. 2002; Fergusson 1999; Fergusson et al. 1993; Maughan et al. 2001; Wakschlag et al. 1997). Kandel et al. (1994) proposed that prenatal exposure to tobacco smoke may affect children’s motivational system. The link between *in utero* exposure to smoking and attention deficit hyperactivity disorder (ADHD) has been demonstrated through meta-analyses of studies published over a 30-year period (Linnet et al. 2003), twin studies (Thapar et al. 2003), as well as studies controlling for socioeconomic status (SES) and pre- and perinatal complications (Batstra et al. 2003a).

Effects of prenatal tobacco smoke exposure on cognitive development in infants are less well understood (Lassen and Oei 1998). Some have suggested that maternal smoking during pregnancy can lead to intellectual delays, most likely caused by central nervous system impairment (Olds et al. 1994), or can negatively affect language ability through underlying physiologic mechanisms (e.g., outer hair cells in the ear), thus leading to poorer performance on language-related tasks (McCartney et al. 1994). Indeed, one of the most consistent neurobehavioral findings is the association between maternal smoking and children’s lower performance on arithmetic and spelling tasks (Batstra et al. 2003a), specific language and auditory tests (Makin et al. 1991; McCartney et al. 1994; Naeye and Peters 1984), reading and language performance (Fried et al. 1997), and verbal learning (Cornelius et al. 2001). Obel and colleagues (1998) observed that smoking ≥10 cigarettes/day during pregnancy was associated with greatly reduced babbling behavior in infants and almost doubled the risk of the infant not becoming a babbler by 8 months of age. This risk was even higher among children who were breast-fed for < 4 months (Obel et al. 1998). Infants born to smoking mothers were reported to have slower auditory habituation, increased sound thresholds, and decreased responsiveness on auditory-related test items at 12 and 24 months of age (Fried and Watkinson 1988). Similarly, children prenatally exposed to smoking scored lower on standardized tests of language development at 3 and 4 years of age (Fergusson et al. 1993). These effects appear to persist though at least 12 years of age (Fried and Watkinson 1988, 2000; Fried et al. 1997, 1998).

The relationship between possible language impairments and maternal smoking has been studied using various types of tests measuring both intellectual and behavioral aspects of development. However, most of the tests can be administered only years after birth, thus allowing for the possibility that other mediating variables contributed to poorer performance instead of prenatal exposure to maternal smoking (e.g., exposure to secondhand smoking during infancy, differences in home environment). In one study, associations between smoking during pregnancy and childhood conduct problems were greatly reduced or eliminated after controlling for antisocial behavior in both parents, depression in mothers, family disadvantage, and genetic influences (Maughan et al. 2004). Environmental exposure to tobacco smoke could be a major contributor to the observed deficits. For example, Makin et al. (1991) noted no clear differences in speech and language ability between the children of mothers who were environmentally exposed to smoking during pregnancy and of those who actually smoked themselves. In a sample of > 3,000 9- to 11-year-olds, children of mothers who quit smoking after delivery scored similar to children of nonsmokers on the Peabody Picture Vocabulary Test and Raven’s Colored Progressive Matrices Test (Bauman et al. 1991). Eskenazi and Castorina (1999) reviewed 17 studies examining effects of maternal smoking during pregnancy and environmental exposure to tobacco, and noted that reported health and cognitive problems in children were often associated more strongly with environmental exposure of the parent and/or child rather than with prenatal smoking exposure alone (e.g., Rantakallio 1983).

One way to control for many of the possible environmental confounds is to investigate the impact of smoking during pregnancy on the infant’s development (including speech processing and other abilities) shortly after birth, when many of the environmental influences are not yet present. The use of event-related potentials (ERPs) to assess the early impact of such exposure offers a way to test infants shortly after birth. The ERP procedure proved to be a valuable tool in gaining insights into cognitive processes of newborns and very young infants who are yet unable to comprehend standard test instructions or provide a reliable behavioral response (e.g., Molfese and Molfese 1979a, 1985, 1997; Molfese et al. 2002; Novak et al. 1989). ERPs are a portion of the ongoing electroencephalogram (EEG) that is time-locked to the onset of a stimulus (e.g., tone, speech sound, or word). Thus, the ERP methodology represents a major advantage over other neurophysiologic measures because it allows researchers to evaluate directly the relationship between a specific event and the resulting brain activity (Callaway et al. 1978; Rockstroh et al. 1982). Additionally, ERPs are advantageous in studying infants’ language development because ERP testing does not require an overt response from the participants and therefore can be conducted within hours after birth, before the possible onset of effects from the postnatal environment. ERPs provide high temporal resolution allowing for millisecond-by-millisecond tracking of the brain’s response to stimulation (e.g., speech syllable), and their spatial resolution is well suited for identifying the scalp patterns and potential underlying sources of brain activity (Molfese et al. 2001, 2002).

Previous studies demonstrated that newborns and young infants are sensitive to differences in vowels (e.g., Cheour et al. 1997; Cheour-Luhtanen et al. 1995; Molfese and Searock 1986) and can differentiate sounds varying in voice onset time (e.g., Molfese and Molfese 1979a; Pang et al. 1998) and place of articulation (Dehaene-Lambertz 2000; Dehaene-Lambertz and Dehaene 1994; Molfese and Molfese 1979b). The latter discrimination, also tested in the present study, was initially evident in the left temporal locations and was followed later by a bilateral effect. Further, infant ERPs in response to speech may reflect the state of an underlying perceptual mechanism that provides a basis for some aspects of verbal and cognitive processes emerging at a later developmental stage. These ERPs therefore can be strongly predictive of performance on language and reading tasks during childhood (Molfese 2000; Molfese and Molfese 1985, 1997). Molfese and Molfese (1985) reported that hemispheric responses at birth differentially sensitive to specific speech stimuli were predictive of children’s later emerging language skills as measured by the McCarthy Scales of Children’s Abilities. Later studies (Molfese 2000; Molfese and Molfese 1997) demonstrated that auditory ERPs to speech sounds recorded at birth discriminate, at well above chance levels, the reading performance in children up to 8 years later. Other researchers have provided converging evidence for the relationship between ERPs in newborns and later language and reading skills (Guttorm et al. 2003; Leppanen et al. 1997; Lyytinen 1997; Lyytinen et al. 2003).

Because smoking leads to adverse intrauterine conditions that may affect development of the auditory perceptual abilities of the exposed infants, it could result in fundamental differences in perceptual skills that set the stage for reported problems in later language outcomes. In turn, language difficulties may contribute to other behavioral and cognitive deficits. For example, Palacios and Semrud-Clikeman (2005) reported a negative linear relationship between reading skills and hyperactivity.

In the current study we examined whether speech-processing ability differs in newborns born to smoking versus nonsmoking mothers. Given the evidence that ERPs reflect hemisphere differences for speech processing early in life (Molfese and Molfese 1979a, 1985; Molfese et al. 1975), we hypothesized that newborns born to smoking mothers would show no hemisphere differences or an altered pattern of such differences compared with the babies of nonsmokers. Second, we expected that newborns exposed to tobacco smoke *in utero* would be less efficient in discriminating speech syllables as indicated by fewer differences in ERPs elicited by different speech syllables.

## Methods

### Participants

We selected 16 neonates (10 females, 6 males) from a larger group of participants included in a prospective study of early language development. This research was reviewed by the University of Louisville Institutional Review Board, and written informed consent was provided by all participants before the study began. All newborns were recruited from the well-baby nursery at a large Midwestern hospital. The participants were between 1–3 days of age (mean ± SD age = 34.56 hours/1.44 days ± 21.36 hours/0.89 days) and were divided into two groups based on prenatal exposure to maternal cigarette smoking. The exposed group included five females and three males (*n* = 8), with a mean age of 48 hours (2 days) ± 18.24 hours (0.76 days). The overall participant pool included additional infants born to mothers who smoked, however, they could not be matched to the controls on some or all of the required criteria, and therefore those data were not included in the analysis. The control group of 5 females and 3 males (*n* = 8), mean age of 21.12 hours (0.88 days) ± 15.36 hours (0.64 days), had no prenatal exposure to maternal smoking, and was matched to the exposed group on sex, birth weight, gestational age, Apgar scores, mother’s age, gravidity, education level, and annual family income (Table 1). A two-tailed *t*-test indicated that the difference of 1 day in the newborns’ mean age was significant (*p* < 0.05); however, the difference is purely accidental and is not related to health differences because all babies were recruited from a well-baby nursery. We obtained maternal smoking history through self-report where mothers identified themselves as smokers or nonsmokers (yes/no) and from medical records available through informed consent. Information about specific amounts of cigarettes smoked was available in the records for five of the eight smoking mothers and indicated mean smoking rates of 11.37 ± 8.33 cigarettes/day.

### Stimuli

The stimuli included six computer-synthesized consonant-vowel (CV) syllables (i.e., /ba/, /da/, /ga/, /bu/, /du/, /gu/), from the “transition-only” stimulus series employed by Stevens and Blumstein (1978). Thus, none of the stimuli contained initial noise bursts. The tokens selected for the present study were the ones most accurately identified by the adult subjects in Stevens and Blumstein’s (1978) study as members of their respective phonetic categories. These were stimulus tokens 1, 7, and 14 from the /ba, da, ga/ continuum, and tokens 1, 7, and 13 from the /bu, du, gu/ continuum, respectively. The five-formant CVs were synthesized so that the amplitude of individual formants was modulated as a function of the respective formant frequencies, as in natural speech. To further improve the naturalness of the stimuli, the vowel /u/ was slightly diphthongized. The central frequencies of the steady-state portion of the formants were kept constant across the different consonants and varied only as a function of the vowel sounds. Duration of F1 transition ranged between 15 and 45 msec across tokens depending on the syllable’s initial consonant as well as on the following vowel. Transition duration for all the other formants was always 40 msec and was followed by a 250-msec steady-state vowel. Rise and decay times were equivalent across sounds.

### Electrodes

A high-density array of 124 silver/silver chloride electrodes embedded in soft sponges (without the lower eye channels; Geodesic Sensor Net, EGI, Inc., Eugene, OR) was used to record the ERPs of the infants. Electrode impedance levels were < 40 kOhm before and after testing; the low pass filter was set to 30 Hz and the high pass filter to 0.1 Hz. During data collection, all electrodes were referred to Cz (vertex) and then were re-referenced offline during data analysis to an average reference.

### Procedure

After permission was obtained from parents, each newborn infant was tested in his or her bassinet in a quiet room in the hospital nursery. During testing, infants were placed on their backs. The high-density array of soft sensors supported infant’s head without creating any localized pressure points, thereby minimizing any potential discomfort. A rolled washcloth was placed under the infant’s neck to further increase comfort. No restraint was used beyond the typical newborn swaddling routinely performed by nurses and parents. The infant’s bassinet was positioned at an angle of approximately 40° to reduce the frequency of alternations in wake–sleep states that are very common in newborn infants. The electrode net was saturated with warm saline solution that acted as a conductor for electrical currents to flow freely from the scalp to the net.

The syllables were presented by a computer at 80 dB SPL(A) (sound pressure level, human hearing range) as measured at the infant’s ear through a speaker positioned approximately 1 m above the midline of the infant’s head. The bassinet was positioned so that the infant’s head was centered below the midline of the speaker to ensure equal speaker-to-ear distance for both ears. All sounds were presented in random order, 25 times each, for a total of 150 trials. Interstimulus intervals varied randomly from 2.5 to 4 sec to prevent habituation. Recording of the brainwaves was controlled by Net Station software (EGI, Inc.). Stimulus presentation was controlled by E-Prime (PST, Inc., Pittsburgh, PA). During the entire test session, the infant’s EEG and behavior were continuously monitored so that the stimulus presentation occurred only when the infant was in a quiet awake state and in proper alignment with the speaker. The recording session lasted approximately 15 min.

### Data analysis

**Figure 1:**
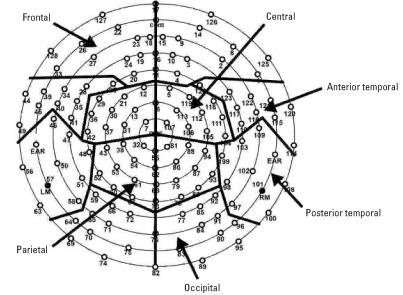
Electrode layout for the 128-channel geodesic sensor net and the channel groups used in the analysis. Abbreviations: com, common; LM, left mastoid; RM, right mastoid.

We obtained individual ERPs by segmenting the ongoing EEG based on each stimulus onset to include a 100-msec prestimulus baseline and a 500-msec post-stimulus interval. ERPs were referenced to an average reference (Junghoefer et al. 1999). Next, artifact rejection was carried out to eliminate ERPs contaminated by movements (e.g., sucking) and eye movement artifacts from further analysis. Rejection rates were comparable across stimulus conditions, with the final averages for each participant including 17–18 trials per condition. Electrodes identified as “bad” (poor signal quality on ≥10% of the trials) were replaced by reconstructing their data using spherical spline interpolation procedures. For a data set to be included in the analyses, a total of no more than 12 channels (10% of the array) could be considered “bad.” Averaged data were then baseline-corrected by subtracting the average microvolt value across the 100-msec prestimulus interval from the poststimulus segment. The 124 electrodes were clustered into 12 regions by averaging the data for electrodes within six regions in the left and right hemispheres respectively: frontal, central, parietal, occipital, anterior temporal, and posterior temporal regions (Figure 1). This reduced the number of variables to increase statistical power. This approach reflected anatomically based boundaries and represented a modification of the clusters used by Key et al. (2006) and Mayes et al. (2005).

Clustered data were submitted to a principal components analysis (PCA) with Varimax rotation using the SPSS version 10 software package (SPSS Inc., Chicago, IL). This process reduced 500 msec of data to a small set of noncorrelated components accounting for the maximum variance. These components corresponded to the areas of maximal variability in the waveform, such as slopes or peaks. Although questions regarding the possibility of misallocation of variance in a PCA analysis across immediately adjacent components have been raised in the past (e.g., Wood and McCarthy 1984), even Wood and McCarthy (1984, p. 258) noted that traditional amplitude and latency approaches are “no less subject to the problem of component overlap” (see also Chapman and McCrary 1995 and Beauducel and Debener 2003 for more recent treatments of this discussion).

The number of factors to be used in later analyses was chosen using the Scree Test (Cattell 1966). Scores from each rotated factor obtained from the PCA served as dependent measures in an analysis of variance (ANOVA) that included one between-group factor—maternal smoking experience—while the remaining factors were repeated across all infants. Thus, the 5-factor design used in the ANOVA was Smoking (2: yes/ no) × Consonant (3: /b,d,g/) × Vowel (2: /a,u/) × Electrode (6: frontal, central, parietal, occipital, anterior temporal, posterior temporal) × Hemisphere (2: left, right). Significant findings were followed by planned comparisons and post hocs.

## Results

The PCA identified three factors that accounted for 93.63% of the total variance. Two of these time intervals reflected group differences and are the focus of this report. Factor 2 accounted for 27.89% of the variance and represented a broad positivity that occurred between 0 and 148 ms. Factor 3 characterized 21.69% of the variance, as a downward (negative) shift between 132 and 268 ms.

**Figure 2:**
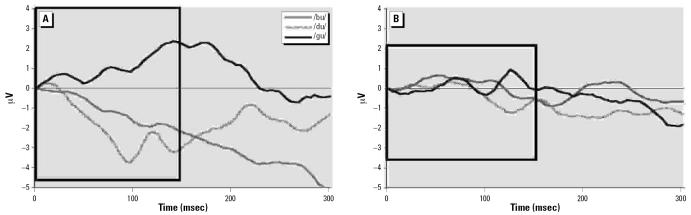
Left posterior temporal ERP differences for consonant sounds for babies of nonsmoking (*A*) and smoking (*B*) mothers. The black box marks the specific time period.

As expected, newborns of smoking and nonsmoking mothers were characterized by significantly different ERPs in response to the speech stimuli. The first 150 msec after stimulus onset were characterized by three interactions involving group differences: Hemisphere × Group, *F*(1,14) = 5.23, *p* < 0.04, power = 0.57, Electrode × Hemisphere × Group, *F*(5,70) = 4.94, *p <* 0.02, power = 0.73, and a Vowel × Consonant × Electrode × Hemisphere × Group interaction *F*(10,140) = 2.66, *p* < 0.05, power = 0.66. Using the smaller interactions as a decision guide, follow-up analyses for the larger interaction were limited to anterior and posterior temporal electrode locations. The results indicated that only the control group discriminated between the different speech syllables in this early time interval (Figure 2). Specifically, over the left posterior temporal locations, the ERPs of newborns born to nonsmokers generated larger, more positive amplitude in response to the /gu/ speech syllable compared with /bu/ and /du/ syllables, [*t* (7) = 2.57, *p* < 0.04 and *t* (7) = 2.71, *p* < 0.03, respectively]. ERPs recorded over right anterior temporal sites differentiated between the /du/ versus /gu/ speech syllables with the former eliciting larger ERP amplitudes, *t* (7) = 3.12, *p* < 0.02. There was also evidence of vowel discrimination at the same locations: /du/ stimuli were characterized by larger amplitude compared with /da/, *t*(7) = 2.81, *p* < 0.03. Additionally, hemisphere asymmetry was observed in response to /gu/ syllables with larger ERP amplitudes occurring over left anterior and posterior temporal sites compared with the homologous sites over the right hemisphere, *t* (7) = 2.81, *p* < 0.03. There were no significant effects for the ERPs obtained from infants born to smokers in this time interval.

**Figure 3:**
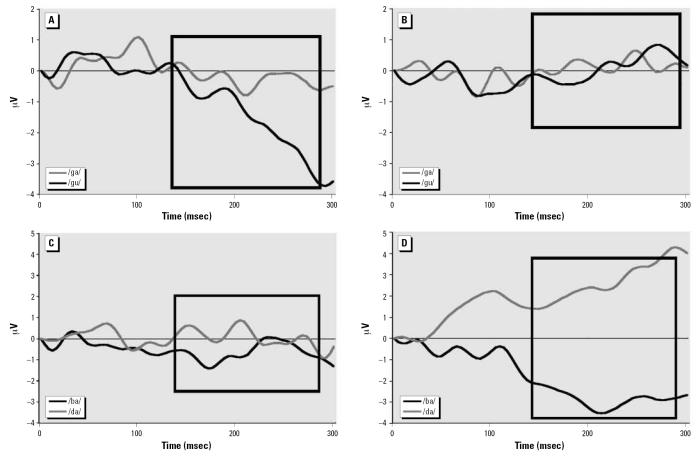
Right posterior temporal ERP differences for vowel (*A*, *B*) and consonant (*C*, *D)* sounds for babies of nonsmoking (*A*, *C*) and smoking (*B*, *D*) mothers. The black box marks the specific time period.

In the second time period identified by the PCA that occurred from 132 msec to 268 msec, babies born to nonsmoking mothers continued to discriminate between speech syllables, whereas infants born to smoking mothers just began to generate ERPs that discriminated between a few of the speech sounds. The babies of nonsmokers discriminated between vowel sounds as indicated by their generating larger ERPs in response to /gu/ compared with /ga/, *t* (7) = 2.46, *p* < 0.05, over right posterior temporal areas (Figure 3A and B) and to /bu/ vs. /ba/, *t* (7) = 3.5, *p* < 0.01, over right parietal regions. Furthermore, the pattern of hemisphere differences was reversed with /gu/ now generating larger amplitudes over right anterior temporal regions rather than left, *t* (7) = 2.53, *p* < 0.04.

In the same time period, ERPs of babies born to smoking mothers generated larger amplitude responses to the /ba/ syllable than to /ga/ over the left posterior temporal sites, *t*(7) = 2.47, *p* < 0.05. This pattern of hemisphere differences was reversed over the right hemisphere where ERP amplitudes to /ba/ were more negative than to /da/, *t* (7) = 2.37, *p* < 0.05 (Figure 3, bottom). Hemisphere asymmetry was present in response to the /ba/ syllable, with larger amplitudes at posterior temporal leads over the left versus right hemisphere, *t* (7) = 3.62, *p* < 0.01. Vowel discrimination was noted for /ba/ versus /bu/ syllables, with the latter eliciting larger amplitudes over right posterior temporal locations, *t* (7) = 2.53, *p* < 0.04. Additionally, these babies generated anterior–posterior temporal differences over the right hemisphere with /da/ eliciting larger amplitudes at posterior locations, *t* (7) = 2.6, *p* < 0.04, an effect that was not present in babies in the nonsmoking group.

## Discussion

Years of medical investigations have demonstrated that smoking during pregnancy adversely affects intrauterine environment of the fetus, leads to lower birth weight, and presents a higher risk for health problems during childhood. Some evidence suggests that prenatal exposure to cigarette smoking results in lower cognitive abilities later in development—specifically, language and reading skills. With one in 10 women in the United States smoking throughout her pregnancy (with individual state rates as high as one in four women; CDC 2004), understanding how prenatal exposure to cigarette smoking affects initial and later cognitive development is crucial.

In this study we examined whether babies born to mothers who smoke process auditory information differently from the babies of non-smokers. ERPs are an effective measure of information processing, have been successfully used in previous studies to assess language processes in newborns and young infants, and have demonstrated strong relationships with the results of standard behavioral assessments. As expected, ERPs elicited by speech stimuli were significantly different in infants prenatally exposed to maternal cigarette smoking. In line with the first hypothesis, newborns of non-smokers demonstrated typical hemisphere asymmetry for speech processing, with larger ERP amplitudes over the left hemisphere than over the right hemisphere—specifically, over anterior and posterior temporal sites. However, babies of smoking mothers initially demonstrated no hemisphere differences, and after a delayed time period their ERPs indicated an inconsistent pattern of hemisphere differences, with left hemisphere amplitudes larger for some speech sounds and smaller for others.

As predicted by the second hypothesis, there were notable group differences in the speed of the brain responses and the number of discriminations between speech sounds. ERPs recorded from babies of nonsmokers began to distinguish consonant and vowel sounds within 150 msec after stimulus onset and discriminated among a larger number of different speech syllables. In contrast, the newborns of smoking mothers began the speech sound differentiation process later in time (after 150 msec) and discriminated among fewer syllables. Together, these findings support the position that smoking during pregnancy has a detrimental effect on the developing fetus expressed in altered brain functioning at birth, even in the absence of low birth weight. Poorer speech sound discrimination at birth could lead to decreased cognitive performance later in life (Molfese 2000). Because this was an observational study, there is an inherent possibility that the group differences might reflect residual confounding by differences between women who smoke and those who do not. However, the use of two groups of healthy newborn infants matched on a variety of maternal and SES characteristics and tested before discharge at the hospital where smoking was not allowed, most of the possible mediating effects (e.g., postnatal environment, smoking-related health issues) were eliminated or greatly reduced, thereby restricting the likely cause of the observed group differences to the prenatal environment.

A markedly delayed speech sound discrimination process observed in newborns of smoking mothers is consistent with previous reports linking prenatal exposure to smoking with less than optimal central nervous system organization and may offer an explanation for lower language abilities during childhood that have been observed in other studies. Electrophysiologic studies report that infants whose brains can detect, react to, and process speech sounds more quickly will be advantaged during later language development (Molfese 2000; Molfese et al. 2002). Similarly, behavioral research supports the notion that phonologic processing skills are fundamental to language development and to subsequent reading abilities (Brady 1991; Catts et al. 1999; Wagner et al. 1994). Others demonstrated a connection between auditory processing and attention deficits (Geffner et al. 1996) or learning disabilities (Kraus et al. 1996). Together, these findings highlight the importance of proper auditory processing and speech discrimination for overall cognitive development and functioning.

One limitation of this study is the minimal information available regarding the exact amount of exposure to tobacco smoke. Maternal smoking histories were obtained through self-report and medical records and did not include detailed information about whether reported smoking rates were stable throughout the pregnancy or other data on environmental exposure to tobacco smoke. Further research is also needed to elucidate the dose–response relationship between the amount of exposure to tobacco smoking *in utero* and the resulting impact on the infant’s brain ability to process speech and other auditory inputs; here, the small number of participants for whom such data were available (*n* = 5) did not allow for sufficient statistical power to examine this issue in the current sample. Using biologic markers (e.g., hair samples, meconium) and including data on environmental exposure to smoking would strengthen future studies by improving the accuracy of classification into exposed/ nonexposed groups and allowing to address dose–response questions.

Furthermore, it is important to examine the longevity of the observed differences in brain activity. A developing brain is known for its plasticity and ability to reorganize in response to stimulation provided by the post-natal environment. For example, breast-feeding may ameliorate some of the negative effects (Batstra et al. 2003b; Obel et al. 1998). Consequently, it is possible that the group differences observed at birth may become smaller over the course of development. However, existing behavioral research reporting group differences between children of smokers and of nonsmokers suggest that, at least in some cases, such differences persist throughout early and middle childhood. Therefore, examining neurodevelopmental trajectories of exposed and nonexposed babies could lead to improved understanding of their differences, better prediction of outcomes, and possibly more specialized and effective interventions designed to compensate for the potential deficits in the exposed group.

Separate consideration should also be given to the environmental exposure to smoking once babies leave the hospital because this may also contribute to the observed developmental differences (Yolton et al. 2005). Infants born to smoking mothers may be more likely to be exposed to environmental tobacco smoke, thus further increasing their risk of adverse developmental outcomes. Finally, although the two participant groups were matched on birth weight, gestational age, mother’s age, Apgar scores, mother’s education, and yearly income, male:female ratio in the current sample was uneven (10 females, 6 males). In the follow-up to this study, it will be important to examine whether smoking during pregnancy differentially affects brain functioning of males and females.

In summary, prenatal exposure to cigarette smoking was found to be associated with suboptimal brain activity related to speech processing in otherwise healthy newborns. A large body of research indicates that ability to differentiate among speech sounds establishes the foundation for later language and cognitive abilities. Although more research is needed, it is clear that smoking during pregnancy places infants at risk for developmental difficulties and thus should be regarded with the attention comparable to that given to other drug use. Further, infants of smoking mothers may benefit from follow-up testing during early childhood that could detect and address any developmental issues as early as possible.

## Figures and Tables

**Table 1:** Participants’ demographic data by group (mean ± SD).

Characteristic	Nonsmokers	Smokers
No. (male/female)	8 (3/5)	8 (3/5)
Gestational age (weeks)	39.5 ± 1.31	39 ± 1.00
Birth weight (g)	3,268.94 ± 277.93	3,426.44 ± 474.20
Mother’s age (years)	25.63 ± 3.78	25.63 ± 4.41
Gravidity	2.75 ± 1.58	2.25 ± 1.04
Apgar scores		
1 min	8.38 ± 0.52	8.38 ± 0.52
5 min	9.00 ± 0.0	8.88 ± 0.35
Family income (thousands, $)	17.81 ± 15.20	17.50 ± 15.47

## References

[b1-ehp0115-000623] American Academy of Pediatrics Committee on Environmental Health (1997). Environmental tobacco smoke: a hazard to children. Pediatrics.

[b2-ehp0115-000623] Batstra L, Hadders-Algra M, Neeleman J (2003a). Effect of antenatal exposure to maternal smoking on behavioural problems and academic achievement in childhood: prospective evidence from a Dutch birth cohort. Early Hum Dev.

[b3-ehp0115-000623] Batstra L, Neeleman J, Hadders-Algra M (2003b). Can breast feeding modify the adverse effects of smoking during pregnancy on the child’s cognitive development?. J Epidemiol Community Health.

[b4-ehp0115-000623] Bauman KE, Flewelling RL, LaPrelle J (1991). Parental cigarette smoking and cognitive performance of children. Health Psychol.

[b5-ehp0115-000623] Beauducel A, Debener S (2003). Misallocation of variance in event-related potentials: simulation studies on the effects of test power, topography, and baseline-to-peak versus principal component quantifications. J Neurosci Methods.

[b6-ehp0115-000623] Brady S 1991. The role of working memory in reading disability. In: Phonological Processes in Literacy: A Tribute to Isabelle Y Liberman (Brady SA, Shankweiler DP, eds). Hillsdale, NJ:Erlbaum, 129–161.

[b7-ehp0115-000623] Brennan PA, Grekin ER, Mortensen EL, Mednick SA (2002). Relationship of maternal smoking during pregnancy with criminal arrest and hospitalization for substance abuse in male and female adult offspring. Am J Psychiatry.

[b8-ehp0115-000623] Callaway E Tueting P Koslow S 1978. Event-Related Brain Potentials in Man. New York:Academic.

[b9-ehp0115-000623] Cattell RB (1966). The Scree Test for the number of factors. Multivariate Behav Res.

[b10-ehp0115-000623] Catts H, Fey M, Zang X, Tomblin J (1999). Language basis of reading and reading disabilities: evidence from a longitudinal investigation. Sci Stud Reading.

[b11-ehp0115-000623] CDC (Centers for Disease Control and Prevention) (2004). Smoking during pregnancy—United States, 1990–2002. Morb Mortal Wkly Rep.

[b12-ehp0115-000623] CDC (Centers for Disease Control and Prevention) (2005). Cigarette smoking among adults—United States, 2004. Morb Mortal Wkly Rep.

[b13-ehp0115-000623] Chapman RM, McCrary JW (1995). EP component identification and measurement by principal components analysis. Brain Cogn.

[b14-ehp0115-000623] Cheour M, Alho K, Sainio K, Reinikainen K, Renlund M, Aaltonen O (1997). The mismatch negativity to changes in speech sounds at the age of 3 months. Dev Neuropsychol.

[b15-ehp0115-000623] Cheour-Luhtanen M, Alho K, Kujala T, Sainio K, Reinikainen K, Renlund M (1995). Mismatch negativity indicates vowel discrimination in newborns. Hear Res.

[b16-ehp0115-000623] Cornelius MD, Ryan CM, Day NL, Goldschmidt L, Willford JA (2001). Prenatal tobacco effects on neuropsychological outcomes among preadolescents. J Dev Behav Pediatr.

[b17-ehp0115-000623] Dehaene-Lambertz G (2000). Cerebral specialization for speech and non-speech stimuli in infants. J Cogn Neurosci.

[b18-ehp0115-000623] Dehaene-Lambertz G, Dehaene S (1994). Speed and cerebral correlates of syllable discrimination in infants. Nature.

[b19-ehp0115-000623] Dejin-Karlsson E, Hanson BS, Ostergren PO, Sjoberg NO, Marsal K (1998). Does passive smoking in early pregnancy increase the risk of small-for-gestational-age infants?. Am J Public Health.

[b20-ehp0115-000623] Ernst M, Moolchan ET, Robinson ML (2001). Behavioral and neural consequences of prenatal exposure to nicotine. J Am Acad Child Adolesc Psychiatry.

[b21-ehp0115-000623] Eskenazi B, Castorina R (1999). Association of prenatal maternal or postnatal child environmental tobacco smoke exposure and neurodevelopmental and behavioral problems in children. Environ Health Perspect.

[b22-ehp0115-000623] Fergusson DM (1999). Prenatal smoking and antisocial behavior. Arch Gen Psychiatry.

[b23-ehp0115-000623] Fergusson DM, Horwood LJ, Lynskey MT (1993). Maternal smoking before and after pregnancy: effects on behavioral outcomes in middle childhood. Pediatrics.

[b24-ehp0115-000623] Fried PA 2002. Tobacco consumption during pregnancy and its impact on child development. In: Encyclopedia on Early Childhood Development. Available: http://www.excellence-earlychildhood.ca/document/FriedANGxp.pdf [accessed 17 July 2005].

[b25-ehp0115-000623] Fried PA, Watkinson B (1988). 12- and 24-month neurobehavioural follow-up of children prenatally exposed to marihuana, cigarettes and alcohol. Neurotoxicol Teratol.

[b26-ehp0115-000623] Fried PA, Watkinson B (2000). Visuoperceptual functioning differs in 9- to 12-year olds prenatally exposed to cigarettes and marihuana. Neurotoxicol Teratol.

[b27-ehp0115-000623] Fried PA, Watkinson B, Dillon RF, Dulberg CS (1987). Neonatal neurological status in a low-risk population after prenatal exposure to cigarettes, marijuana, and alcohol. J Dev Behav Pediatr.

[b28-ehp0115-000623] Fried PA, Watkinson B, Gray R (1998). Differential effects on cognitive functioning in 9- to 12-year olds prenatally exposed to cigarettes and marihuana. Neurotoxicol Teratol.

[b29-ehp0115-000623] Fried PA, Watkinson B, Siegel LS (1997). Reading and language in 9- to 12-year olds prenatally exposed to cigarettes and marijuana. Neurotoxicol Teratol.

[b30-ehp0115-000623] Geffner D, Lucker JR, Koch W (1996). Evaluation of auditory discrimination in children with ADD and without ADD. Child Psychiatry Hum Dev.

[b31-ehp0115-000623] Guttorm TK, Leppanen PH, Tolvanen A, Lyytinen H (2003). Event-related potentials in newborns with and without familial risk for dyslexia: principal component analysis reveals differences between the groups. J Neural Transm.

[b32-ehp0115-000623] Hardy JB, Mellits ED (1972). Does maternal smoking during pregnancy have a long-term effect on the child?. Lancet.

[b33-ehp0115-000623] Hellstrom-Lindahl E, Nordberg A (2002). Smoking during pregnancy: a way to transfer the addiction to the next generation?. Respiration.

[b34-ehp0115-000623] Junghoefer M, Elbert T, Tucker D, Braun C (1999). The polar effect of average reference: a bias in estimating the head surface intergal in EEG recording. Electroencephalogr Clin Neurophysiol.

[b35-ehp0115-000623] Kandel DB, Wu P, Davies M (1994). Maternal smoking during pregnancy and smoking by adolescent daughters. Am J Public Health.

[b36-ehp0115-000623] Key A, Molfese DL, Ratajczak E (2006). ERP indicators of learning in adults. Dev Neuropsychol.

[b37-ehp0115-000623] Kraus N, McGee TJ, Carrell TD, Zecker SG, Nicol TG, Koch DB (1996). Auditory neurophysiologic responses and discrimination deficits in children with learning problems. Science.

[b38-ehp0115-000623] Lassen K, Oei TP (1998). Effects of maternal cigarette smoking during pregnancy on long-term physical and cognitive parameters of child development. Addict Behav.

[b39-ehp0115-000623] Law KL, Stroud LR, LaGasse LL, Niaura R, Liu J, Lester BM (2003). Smoking during pregnancy and newborn neurobe-havior. Pediatrics.

[b40-ehp0115-000623] Leppanen PHT, Eklund KM, Lyytinen H (1997). Event-related brain potentials to change in rapidly presented acoustic stimuli in newborns. Dev Neuropsychol.

[b41-ehp0115-000623] Linnet KM, Dalsgaard S, Obel C, Wisborg K, Henriksen TB, Rodriguez A (2003). Maternal lifestyle factors in pregnancy risk of attention deficit hyperactivity disorder and associated behaviors: review of the current evidence. Am J Psychiatry.

[b42-ehp0115-000623] Longo LO (1977). The biological effects of carbon monoxide on the pregnant woman, fetus, and newborn infant. Am J Obstet Gynecol.

[b43-ehp0115-000623] Lyytinen H 1997. In search of precursors of dyslexia: a prospective study of children at risk for reading problems. In: Dyslexia: Biology, Cognition and Intervention (Snowling M, ed). London:Whurr Publishers, 97–107.

[b44-ehp0115-000623] Lyytinen H Leppanen PH Richardson U Guttorm TK 2003. Brain functions and speech perception in infants at risk for dyslexia. In: Dyslexia: Different Brain, Different Behaviour (Csépe V, ed). Dordrecht:Kluwer, 113–152.

[b45-ehp0115-000623] Makin J, Fried PA, Watkinson B (1991). A comparison of active and passive smoking during pregnancy: long-term effects. Neurotoxicol Teratol.

[b46-ehp0115-000623] Maughan B, Taylor A, Caspi A, Moffitt TE (2004). Prenatal smoking and early childhood conduct problems: testing genetic and environmental explanations of the association. Arch Gen Psychiatry.

[b47-ehp0115-000623] Maughan B, Taylor C, Taylor A, Butler N, Bynner J (2001). Pregnancy smoking and childhood conduct problems: a causal association?. J Child Psychol Psychiatry.

[b48-ehp0115-000623] Mayes L, Molfese DL, Key A, Hunter N (2005). Event-related potentials in cocaine-exposed children during a Stroop task. Neurotoxicol Teratol.

[b49-ehp0115-000623] McCartney JS, Fried PA, Watkinson B (1994). Central auditory processing in school-age children prenatally exposed to cigarette smoke. Neurotoxicol Teratol.

[b50-ehp0115-000623] Molfese DL (2000). Predicting dyslexia at 8 years of age using neonatal brain responses. Brain Lang.

[b51-ehp0115-000623] Molfese DL, Freeman RB, Palermo DS (1975). The ontogeny of brain lateralization for speech and nonspeech stimuli. Brain Lang.

[b52-ehp0115-000623] Molfese DL, Molfese VJ (1979a). Hemisphere and stimulus differences as reflected in the cortical responses of newborn infants to speech stimuli. Dev Psychol.

[b53-ehp0115-000623] Molfese DL Molfese VJ 1979b. Infant speech perception: Learned or innate. In: Advances in Neurolinguistics (Whitaker HW, Whitaker HA, ed). New York:Academic Press, 225–240.

[b54-ehp0115-000623] Molfese DL, Molfese VJ (1985). Electrophysiological indices of auditory discrimination in newborn infants: The bases for predicting later development?. Infant Behav Dev.

[b55-ehp0115-000623] Molfese DL, Molfese VJ (1997). Discrimination of language skills at five years of age using event-related potentials recorded at birth. Dev Neuropsychol.

[b56-ehp0115-000623] Molfese DL, Molfese VJ, Kelly S (2001). The use of brain electro-physiology techniques to study language: a basic guide for the beginning consumer of electrophysiology information. Learn Disability Q.

[b57-ehp0115-000623] Molfese DL, Molfese VJ, Key A, Modglin A, Kelly S, Terrel S (2002). Reading and cognitive abilities: Longitudinal studies of brain behavior and changes in young children. Interdiscip J Int Dyslexia Assoc.

[b58-ehp0115-000623] Molfese DL, Searock KJ (1986). The use of auditory evoked responses at one-year-of-age to predict language skills at 3-years. Aust J Commun Disorders.

[b59-ehp0115-000623] Naeye RL, Peters EC (1984). Mental development of children whose mothers smoked during pregnancy. Obstet Gynecol.

[b60-ehp0115-000623] Novak GP, Kurtzberg D, Kreuzer JA, Vaughan HG (1989). Cortical responses to speech sounds and their formants in normal infants: maturational sequence and spatiotemporal analysis. Electroencephalogr Clin Neurophysiol.

[b61-ehp0115-000623] Obel C, Henriksen TB, Hedegaard M, Secher NJ, Ostergaard J (1998). Smoking during pregnancy and babbling abilities of the 8-month-old infant. Paediatr Perinat Epidemiol.

[b62-ehp0115-000623] Olds DL, Henderson CR, Tatelbaum R (1994). Prevention of intellectual impairment in children of women who smoke cigarettes during pregnancy. Pediatrics.

[b63-ehp0115-000623] Oliff HS, Gallardo KA (1999). The effect of nicotine on developing brain catecholamine systems. Front Biosci.

[b64-ehp0115-000623] Palacios ED, Semrud-Clikeman M (2005). Delinquency, hyperactivity, and phonological awareness: a comparison of adolescents with ODD and ADHD. Appl Neuropsychol.

[b65-ehp0115-000623] Pang EW, Edmonds GE, Desjardins R, Khan SC, Trainor LJ, Taylor MJ (1998). Mismatch negativity to speech stimuli in 8-month-old infants and adults. Int J Psychophysiol.

[b66-ehp0115-000623] Rantakallio P (1983). A follow-up study up to the age of 14 of children whose mothers smoked during pregnancy. Acta Paediatr Scand.

[b67-ehp0115-000623] Rockstroh B Elbert T Birbaumer N Lutzenberger W 1982. Slow Brain Potentials and Behavior. Baltimore:Urban-Schwarzenberg.

[b68-ehp0115-000623] Roy TS, Sabherwal U (1994). Effects of prenatal nicotine exposure on the morphogenesis of somatosensory cortex. Neurotoxicol Teratol.

[b69-ehp0115-000623] Slotkin TA (1998). Fetal nicotine or cocaine exposure: which one is worse?. J Pharmacol Exp Ther.

[b70-ehp0115-000623] Stevens KN, Blumstein SE (1978). Invariant cues for place of articulation in stop consonants. J Acoust Soc Am.

[b71-ehp0115-000623] Thapar A, Fowler T, Rice F, Scourfield J, van den Bree M, Thomas H (2003). Maternal smoking during pregnancy and attention deficit hyperactivity disorder symptoms in offspring. Am J Psychiatry.

[b72-ehp0115-000623] Wagner R, Torgesen J, Raschotte C (1994). Development of reading-related phonological processing abilities: new evidence of bidirectional causality from a latent variable longitudinal study. Dev Psychol.

[b73-ehp0115-000623] Wakschlag LS, Hans SL (2002). Maternal smoking during pregnancy and conduct problems in high-risk youth: a developmental framework. Dev Psychopathol.

[b74-ehp0115-000623] Wakschlag LS, Lahey BB, Loeber R, Green SM, Gordon RA, Leventhal BL (1997). Maternal smoking during pregnancy and the risk of conduct disorder in boys. Arch Gen Psychiatry.

[b75-ehp0115-000623] Weitzman M, Byrd RS, Aligne CA, Moss M (2002). The effects of tobacco exposure on children’s behavioral and cognitive functioning: implications for clinical and public health policy and future research. Neurotoxicol Teratol.

[b76-ehp0115-000623] Wood CC, McCarthy G (1984). Principal component analysis of event-related potentials: simulation studies demonstrate misallocation of variance across components. Electro-encephalogr Clin Neurophysiol.

[b77-ehp0115-000623] Yolton K, Dietrich K, Auinger P, Lanphear B, Hornung R (2005). Exposure to environmental tobacco smoke and cognitive abilities among U.S. children and adolescents. Environ Health Perspect.

